# Deep learning-based automated segmentation of eight brain anatomical regions using head CT images in PET/CT

**DOI:** 10.1186/s12880-022-00807-4

**Published:** 2022-05-26

**Authors:** Tong Wang, Haiqun Xing, Yige Li, Sicong Wang, Ling Liu, Fang Li, Hongli Jing

**Affiliations:** 1 Department of Nuclear Medicine, Peking Union Medical College Hospital, Beijing, China; 2 GE Healthcare China, Shanghai, China

**Keywords:** PET/CT, CT, Brain segmentation, Deep learning, MRI, CNN

## Abstract

**Objective:**

We aim to propose a deep learning-based method of automated segmentation of eight brain anatomical regions in head computed tomography (CT) images obtained during positron emission tomography/computed tomography (PET/CT) scans. The brain regions include basal ganglia, cerebellum, hemisphere, and hippocampus, all split into left and right.

**Materials and methods:**

We enrolled patients who underwent both PET/CT imaging (with an extra head CT scan) and magnetic resonance imaging (MRI). The segmentation of eight brain regions in CT was achieved by using convolutional neural networks (CNNs): DenseVNet and 3D U-Net. The same segmentation task in MRI was performed by using BrainSuite13, which was a public atlas label method. The mean Dice scores were used to assess the performance of the CNNs. Then, the agreement and correlation of the volumes of the eight segmented brain regions between CT and MRI methods were analyzed.

**Results:**

18 patients were enrolled. Four of the eight brain regions obtained high mean Dice scores (> 0.90): left (0.978) and right (0.912) basal ganglia and left (0.945) and right (0.960) hemisphere. Regarding the agreement and correlation of the brain region volumes between two methods, moderate agreements were observed on the left (ICC: 0.618, 95% CI 0.242, 0.835) and right (ICC: 0.654, 95% CI 0.298, 0.853) hemisphere. Poor agreements were observed on the other regions. A moderate correlation was observed on the right hemisphere (Spearman’s rho 0.68, p = 0.0019). Lower correlations were observed on the other regions.

**Conclusions:**

The proposed deep learning-based method performed automated segmentation of eight brain anatomical regions on head CT imaging in PET/CT. Some regions obtained high mean Dice scores and the agreement and correlation results of the segmented region volumes between two methods were moderate to poor.

## Introduction

Positron emission tomography/computed tomography (PET/CT) provides both functional and anatomical information of the human body in a single scan and has been widely used in clinical practice [1, 2]. PET imaging extracts the metabolic information of the body with ^18^F-fluorodeoxy-glucose (FDG) while CT imaging captures the anatomical information since it has a higher spatial resolution than PET. For the application of brain segmentation, using PET-only images is challenging because of the low spatial resolution and high noise level in PET data [3]. A few fusion methods have been proposed to combine the complementary information from PET/CT to perform brain tissue segmentation [2–5]. However, one downside of these fusion methods is the unreasonable use of the inconsistent information from different imaging modalities [1]. Thus, how to take advantage of the complementary information from PET/CT remains to be explored.

Compared to using PET-only images or PET/CT images, brain segmentation in magnetic resonance imaging (MRI) or CT has been more widely studied and MRI has always been used because of its superior soft tissue contrast [6]. There are various segmentation tasks and objectives, e.g., the classification of white matter (WM), grey matter (GM), and cerebrospinal fluid (CSF) [7]. There are also studies that focus on certain pathologies, such as neonatal brain development [8–10], traumatic brain injury (TBI) [11], and brain tumor segmentation [12]. Deep learning methods have gained increasing attention in the field in the past few years [13, 14]. For clinical scenarios where MRI cannot be performed, e.g., emergency situations, patients with metal implants or claustrophobia, and cost issues, CT imaging can be an alternative modality. The advantages of CT over MRI include faster acquisition, lower cost, and wide availability.

However, studies on brain segmentation in CT have been sparse compared to those in MRI. A systematic review by Lenchik et al. [15] showed that 94% of the neurologic segmentation studies were using MRI and only 5% were using CT. Wang et al. [16] applied sparse representation techniques and proposed a novel patch-driven level set method for neonatal brain segmentation in MRI. Zhang et al. [17] proposed to use deep convolutional neural networks for isointense stage brain tissue segmentation using multi-modality MRI. More recently, Zhang et al. [18] proposed a novel task-structured brain tumor segmentation network (TSBTS net) to perform brain tumor segmentation. Besides, a novel cross-modality deep feature learning framework [19] was proposed to segment brain tumor by taking advantage of mining rich patterns across the multi-modality data. Despite the limited numbers, Hu et al. [20] proposed an algorithm to determine WM and GM from CT head volumes with large slice thickness based on thresholding and brain mask propagation. Lee et al. [21] presented the combination of different approaches for the segmentation of abnormal regions, CSF, and brain matter. Manniesing et al. [22] presented an automated segmentation method to classify WM and GM in contrast-enhanced 4D CT images. More recently, Qian et al. [23] presented an active contour model for the segmentation of CSF in CT images. With the advances of deep learning and machine learning methods, Zhao et al. [6] proposed using a deep learning method to synthesize MR images from CT images and then used the synthetic MR images for whole brain segmentation and labeling. Cai et al. [24] developed a deep learning model that performed segmentation of intracranial structures on head CT images.

Previous approaches primarily focused on the segmentation of three brain tissue types: WM, GM, and CSF [20–22]. However, there are considerably more brain tissue types and brain anatomical regions or structures that are of physiological and pathological significance, such as cerebellum, brain stem, and basal ganglia [25]. Even though only a limited number of studies performing brain segmentation in CT using deep learning methods have been published, they showed promising results.

In our study, we aim to propose a deep learning-based segmentation method in CT to classify eight brain tissue regions: basal ganglia, cerebellum, hemisphere, and hippocampus, all split into left and right. We use deep neural networks to perform the task. Although there have been more efforts into the brain segmentation with CT methods [20–24, 26, 27], we could not find a public and well-established CT atlas-based method or tool to serve as the comparison reference. Thus, results are compared to those obtained in MRI images with a conventional atlas-label method. The atlas-based method in BrainSuite13 [30] has been a well-established, widely used tool in the field and it can segment the same eight brain anatomical regions as our proposed method does, which qualifies to be the comparison reference. Also, the comparison between CT and MRI has been conducted in previous studies [6, 27]. The additional systematic error due to the registration error between CT and MRI is a topic that is worth further investigation. However, it is not within the scope of this study.

## Materials and methods

### Patients and imaging protocol

We retrospectively enrolled patients who underwent both PET/CT and MRI at the Peking Union Medical College Hospital. The PET/CT scan was performed on a Siemens BioGraph PET/CT scanner (Siemens Healthineers, Erlangen, Germany). The whole-body PET/CT scanning protocol was described in a previous study [28]. A separate head CT imaging was also performed, and a full head coverage from vertex to skull base was achieved. The voltage output of the X-Ray generator was 120 kVp and the X-Ray tube current was 300 mAs. The head CT images had the voxel spacing (resolution) of 0.6 × 0.6 × 1.5 mm^3^ with dimensions of 512 × 512 × 148. The MRI scan was performed on a Toshiba Vantage Titan 3 T scanner (Canon Medical Systems, Tochigi, Japan). Both T1-weighted (T1-w) and T2-weighted (T2-w) MR images were acquired. T1-w images were obtained using the sequence with the following parameters: TR 2100 ms, TE 10 ms, TI 900 ms. T2-w images used these parameters: TR 4650 ms, TE 95 ms. For both T1-w images and T2-w images, the dimensions were 640 × 640 × 24 and the voxel spacing was 0.36 × 0.36 × 6 mm^3^. During post-processing, both CT images and MR images were reconstructed to have the same resolution of 1 × 1 × 2 mm^3^, resulting in a single voxel volume of 2 mm^3^.

### MRI segmentation with an atlas label method

Shattuck et al. [29] proposed a new MRI analysis tool, BrainSuite, that produced cortical surface representation with spherical topology from human head MR images. The tool could perform accurate brain segmentations in a single package based on a sequence of low-level operations. The operations included skull and scalp removal, image nonuniformity compensation, voxel-based tissue classification, topological correction, rendering, and editing functions. Later, Shattuck et al. [30] proposed BrainSuite13, a collection of software tools for jointly processing and visualizing structural and diffusion MRI of the human brain.

In our study, we used BrainSuite13 to perform the brain segmentation task in MRI. First, full-head T1-w MR images were processed to achieve automated cortical surface extraction. Then, the generated cortical mesh models were registered spatially to a labeled brain atlas, which included eight different brain anatomical structures, i.e., hemisphere, hippocampus, basal ganglia, and cerebellum, all split into left and right. The atlas was from a single subject and the registration was performed using a combined surface/volume procedure [31]. After the registration, the labels of the surface and volume were transferred from the atlas to the subject, segmenting the subject MRI into the delineated region of interest (ROI). For the ROI boundaries to conform to the bottoms of the sulcal valleys, cortical surfaces were refined locally at the mid-cortical surface using geodesic curvature flow [32].

### CT segmentation with convolutional neural networks

In this study, we utilized two convolutional neural networks (CNNs) (the DenseVNet and the 3D U-Net) to accomplish the segmentation of brain anatomical regions in CT. First, 90 patients with non-contrast computed tomography (NCCT) images were enrolled. The CNNs were trained and tested on this 90-patient data set. Then, the CT images of 18 patients, whose acquisition details were described in chapter 2.1, were used as an independent testing data set. Later, the segmentation results obtained on this 18-patient CT data set with the trained CNN model, in addition to the MRI segmentation results of the same 18 patients, were used to conduct head-to-head volumetric comparisons. The trained CNN was embedded into the NovoStroke Kit (NSK) software (research-only prototype, GE Healthcare, China). The details of data acquisition, data preprocessing, and model training and testing are discussed below.

#### Data acquisition

To train the CNNs, 90 patients were enrolled from two separate stroke centers. All enrolled patients underwent both non-contrast computed tomography (NCCT) and computed tomography perfusion (CTP). NCCT images were used for the brain segmentation task. 44 NCCT datasets from center A were acquired on a GE Revolution CT scanner (voltage: 120 kVp, current: 225 mAs) with a voxel spacing of 0.5 × 0.5 × 2.5 mm^3^ and dimensions of 512 × 512 × 64. 46 datasets from center B were acquired on a GE Revolution CT scanner (voltage: 120 kVp, current: 174 mAs) with a voxel spacing of 0.5 × 0.5 × 5 mm^3^ and dimensions of 512 × 512 × 32. 90 patients were split into the training set with 81 patients and the testing set with 9 patients.

The ground truth was defined by manual annotation by a neuroradiologist with more than 20 years of experience. Each axial slice was annotated, resulting in a segmentation of eight brain anatomical regions: basal ganglia, cerebellum, hemisphere, and hippocampus, all split into left and right. The same regions were segmented in the MRI atlas method. The annotation was performed by using the Medical Imaging Interaction Toolkit 2018 (MITK 2018) software.

#### Data preprocessing

Before training and testing, all 90 datasets were preprocessed by several operations. Firstly, all 3D image data were resampled to obtain the same voxel spacing of 0.5 × 0.5 × 5 mm^3^ by linear interpolation. Secondly, a Gaussian filter with sigma = 0.5 was utilized to remove the noise in CT images. Thirdly, we used skull-stripping to eliminate the skull region of the head so only the soft brain tissues remained [33]. Finally, the brain parenchyma was refined by threshold method. All CT values which were not in the range of [0, 120] were reset to zero.

#### Model training and testing

**Fig. 1:**
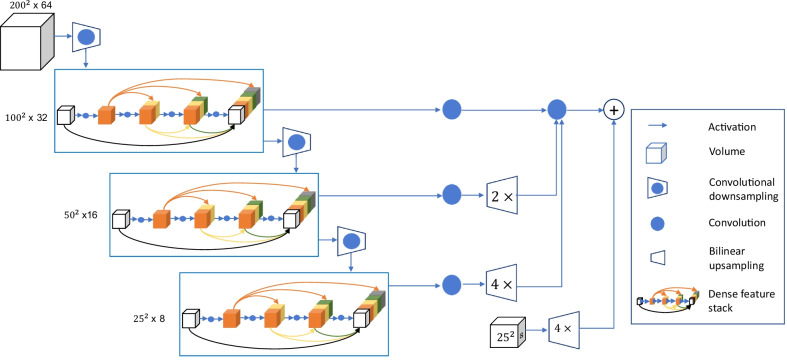
Convolutional neural network, DenseVNet, used in this study. It consisted of 5 key features, including batch-wise spatial dropout, dense feature stacks, V-network downsampling and upsampling, dilated convolution, and an explicit spatial prior

The CNNs used were the DenseVNet [34] and 3D U-Net [35]. Figure 1 shows the network architecture of the DenseVNet used in our study. It consisted of 5 key features, including batch-wise spatial dropout, dense feature stacks, V-network downsampling and upsampling, dilated convolution, and an explicit spatial prior. Structure of the 3D U-Net can be found in [35].

We trained the network on the framework of Niftynet 1.0.4 version. The graphic card used for training was NVIDIA Quadro P3200, which had 8 GB memory. The spatial window size was set as 200 × 200 × 64 for DenseVNet and as 96 × 96 × 96 for 3D U-Net. The window size was kept the same for training and testing for both network models. Also, the batch size was set as 1 for both network models. In addition, we chose the Adam gradient descent algorithm to reduce the training errors. For the first 2000 epochs, we chose 0.001 as the learning rate and 50 datasets for training. In the next 2000 epochs, we adapted the learning rate as 0.00025 and used the rest 31 datasets for training. The whole training costed nearly 7 h for DenseVNet and 4 h for 3D U-Net. This strategy was used for both networks and we used it to elevate the performance of segmenting the left and right hippocampus. A comparative experiment (on DenseVNet only) was conducted where the performance was compared between this strategy and the one using 4000 epochs and the constant learning rate of 0.001. The performance of CNN was evaluated by using the Dice similarity score [36].

### Evaluation of the CT segmentation method and volumetric comparison to MRI results

We assessed the performance of the CT segmentation method by using an independent testing set, which was described in the patients and imaging protocol section. Performance metrics identical to those during the training and testing phase were recorded. The CNN with superior results would be used as the CT segmentation method for the following experiments. Since enrolled patients were required to undergo both MRI and PET/CT, we then performed a head-to-head comparison of the voxel volumes of the segmented brain structures between the CT method and the MRI method. The MRI results were used as references. Correlation was assessed by conducting non-parametric correlation (Spearman’s rho). For interpreting correlation coefficients, values less than 0.4, between 0.4 and 0.7, between 0.7 and 0.9, and greater than 0.9 are indicative of weak, moderate, strong, and very strong correlation, respectively [37]. Intraclass correlation (ICC) was calculated to assess the agreement based on a two-way mixed, absolute agreement, single measures model [38]. Based on the 95% confident interval (CI), values less than 0.5, between 0.5 and 0.75, between 0.75 and 0.9, and greater than 0.9 are indicative of poor, moderate, good, and excellent reliability, respectively [38]. Besides, Student’s t test or the Wilcoxon Rank Sum Test was also conducted to verify the difference between two methods, depending on if the data were normally distributed.

Statistical analysis was performed on MedCalc 19.1 (Ostend, Belgium). Statistical significance was considered for a p value less than 0.05.

## Results

### Performance of CNNs during training and testing process

**Fig. 2:**
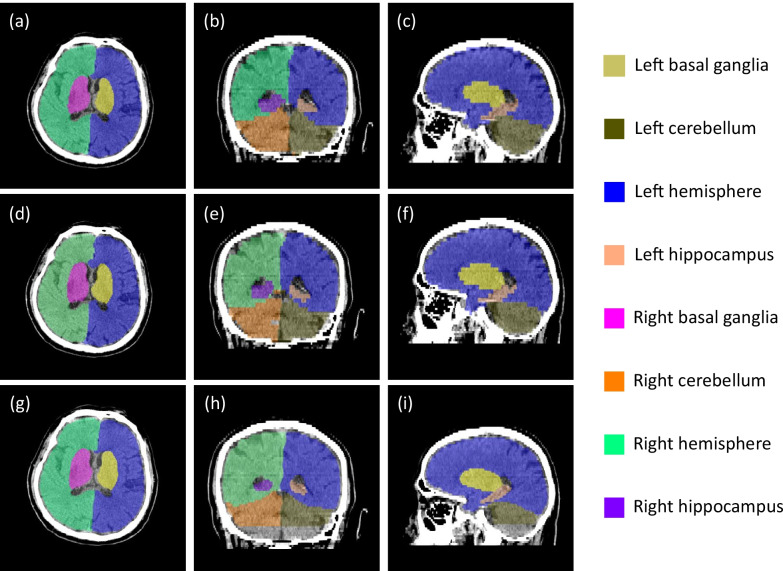
Representative segmentation results of one subject from the testing set. **a** Axial, **b** coronal, and **c** sagittal section of the CT images with labels obtained from the DenseVNet model. **d** Axial, **e** coronal, and **f** sagittal section of the CT images with ground truth labels. **g** Axial, **h** coronal, and **i** sagittal section of the CT images with labels obtained from the 3D U-Net model. Eight brain anatomical regions (all split into left and right): basal ganglia, cerebellum, hemisphere, and hippocampus are color-coded and marked on the right

Figure 2 shows representative segmentation results of eight brain anatomical regions of one subject from the testing set. Figure 2a–c shows the axial, coronal, and sagittal section of the CT images with segmentation labels obtained from the DenseVNet model, respectively. Figure 2d–f show the same sections of the same subject with labels of the ground truth. Figure 2g–i show the results from the 3D U-Net model. Eight regions were color-coded and marked on the right side of the figure. The results showed overall high consistency by naked eye between the DenseVNet model and the ground truth. The results of the 3D U-Net model showed overall consistency to the ground truth, but we can observe some missed labels at the bottom of Fig. 2h, i. Table 1 shows the mean Dice scores obtained during the training and testing process. The results of comparing two strategies for DenseVNet are shown. One the testing set, we can see the Dice scores of the left and right hippocampus with the varying learning rate strategy was increased from 0.676 to 0.711 and 0.743 to 0.758, respectively. Meanwhile, the performance on the other regions were almost equivalent. For comparing the results of two CNNs, DenseVNet obtained better results than 3D U-Net did. For the training set with DenseVNet, all Dice scores were larger than 0.90 except for left and right hippocampus. However, with 3D U-Net, Dice scores were larger than 0.90 only in left and right hemisphere. For the testing set with DenseVNet, all Dice scores were larger than 0.90 except for left and right hippocampus, and right cerebellum. However, with 3D U-Net, all Dice scores were smaller than 0.90.

**Table 1:** Dice scores of eight brain anatomical regions during the training and testing process with different deep learning models

Dice scores								
	Basal ganglia		Cerebellum		Hemisphere		Hippocampus	
	Left	Right	Left	Right	Left	Right	Left	Right
*Training*								
DenseVNet 4000 epochs, LR = 0.001	0.944	0.932	0.939	0.919	0.963	0.961	0.872	0.864
DenseVNet 2000 epochs, LR = 0.001 2000 epochs, LR = 0.00025	0.943	0.937	0.938	0.917	0.963	0.960	0.888	0.854
3D U-Net	0.855	0.755	0.802	0.797	0.921	0.915	0.723	0.571
*Testing*								
DenseVNet 4000 epochs, LR = 0.001	0.927	0.908	0.911	0.892	0.957	0.956	0.676	0.743
DenseVNet 2000 epochs, LR = 0.001 2000 epochs, LR = 0.00025	0.926	0.912	0.909	0.884	0.956	0.953	0.711	0.758
3D U-Net	0.891	0.745	0.808	0.812	0.866	0.839	0.544	0.565

*LR *learning rate

### Patient demographics

In total, 18 patients who underwent PET/CT, head CT, and MRI were enrolled (10 women, 8 men, age: 50 ± 13.7 (mean ± SD) years, minimum age 21, maximum age 70). From the PET/CT scans, 5 patients showed no abnormal metabolic activity on the brain. 13 patients showed certain levels of abnormal metabolic activity. However, brain anatomical structures were examined by the neuroradiologist to make sure segmentation could be performed.

### Segmentation results of CT CNNs and MRI atlas method

**Fig. 3:**
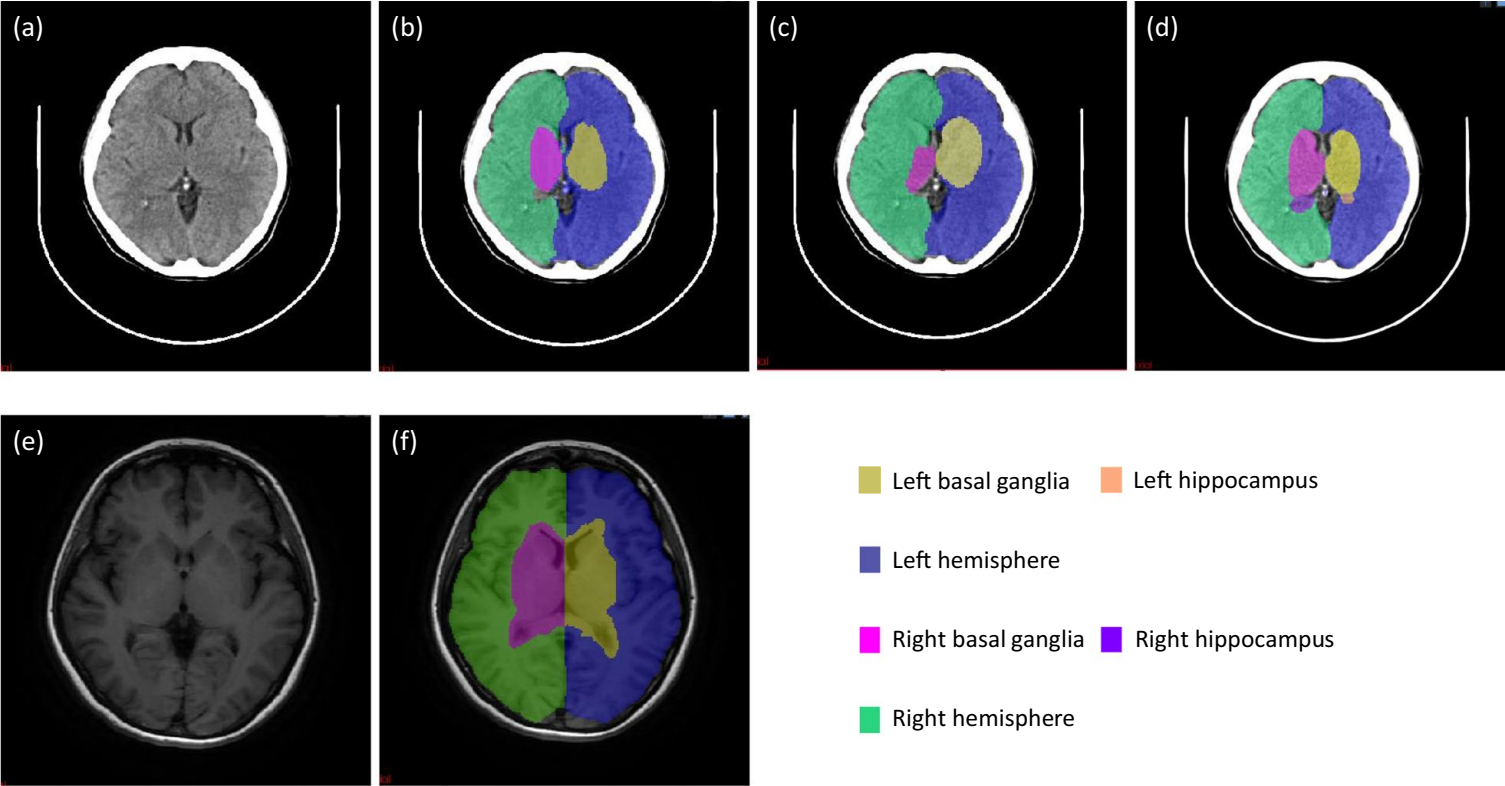
Representative segmentation results of one subject from the independent testing set, including **a** the input CT image, **b** the ground truth labels with the input CT image, **c** the segmentation labels of DenseVNet with the input CT image, **d** the segmentation labels of 3D U-Net with the input CT image, **e** the input MRI image, and **f** the segmentation labels of the MR atlas method with the MRI image

Figure 3 shows the representative segmentation results of one subject from the independent testing set, including (a) the input CT image, (b) the ground truth labels with the input CT image, (c) the segmentation labels of DenseVNet with the input, and (d) the segmentation labels of 3D U-Net with the input. (e) The input MRI image and (f) the segmentation labels of the MR atlas method are also included. Overall, the segmentation labels shown in Fig. 3c, d, f indicated that the left and right hemisphere matched well between different methods. However, there were observable differences on the basal ganglia and hippocampus regions. Firstly, the differences might be due to the inherent distinction between the ground truth in CT and that in MRI. Secondly, by comparing Fig. 3b–d, we observed that the segmentation using CNNs introduced variations as well.

The mean Dice scores on the 18 subjects are shown in Table 2. It should be emphasized that this was the independent testing set so the models had not seen these data before. Again, DenseVNet obtained better results than 3D U-Net did. Thus, the trained DenseVNet model was used as the CT segmentation method for the following head-to-head volumetric comparisons. To look at the results with DenseVNet, four anatomical regions including left and right basal ganglia, left and right hemisphere obtained high Dice scores (> 0.90). Other regions including left and right cerebellum, left and right hippocampus obtained low Dice scores, especially left hippocampus.

**Table 2:** Dice scores of eight brain anatomical regions on the independent testing data set of 18 subjects with two different deep learning models

Dice scores								
	Basal ganglia		Cerebellum		Hemisphere		Hippocampus	
	Left	Right	Left	Right	Left	Right	Left	Right
*Independent testing*								
DenseVNet	0.978	0.912	0.689	0.867	0.945	0.960	0.089	0.32
3D U-Net	0.524	0.272	0.524	0.612	0.593	0.506	0.089	0.079

### Agreement and correlation of volume results between CT and MRI segmentations

Both CT and MRI brain segmentation methods yielded voxel-based volume results on each brain region. The voxel-based volume distributions, performance of ICC, and correlations between two methods are summarized in Table 3. For voxel-based volume results, mean ± standard deviation (SD) was shown if the data were normally distributed. And median (quartile 1, quartile 3) was shown if the data were not normally distributed. There was no statistical difference of the voxel-based volume of each region between two methods, except for left basal ganglia (p < 0.05). The ICC between two methods was moderate for left and right Hemisphere (ICC: 0.618, 95% CI 0.242, 0.835; and ICC: 0.654, 95% CI 0.298, 0.853). Lower ICC results were observed for the other regions, especially the right hippocampus. In terms of the correlation, we found that left hemisphere (Spearman’s rho 0.591; p = 0.0097), left hippocampus (Spearman’s rho 0.564; p = 0.0147) and right hemisphere (Spearman’s rho 0.68; p = 0.0019) obtained moderate correlation between CT and MRI methods and were statistically significant. No significant correlation was found in the other regions (p > 0.05).

**Table 3:** Agreement and correlation results of the voxel-based volumes by CT and MRI methods

Brain regions	CT voxel-based volume	MR voxel-based volume	p value	ICC	Spearman's coefficient	Correlation p value
Left basal ganglia	45,804.50 (44,319.00, 47,000.00)	48,837.00 (45,742.50, 52,271.00)	0.011	0.315 (− 0.097 to 0.662)	0.39 (− 0.094 to 0.725)	0.109
Left cerebellum	89,802.78 ± 5571.08	92,617.67 ± 5391.20	0.133	0.362 (− 0.059 to 0.69)	0.277 (− 0.218 to 0.659)	0.265
Left hemisphere	531,664.00 (483,331.80, 550,391.80)	504,269.00 (480,361.10, 539,919.80)	0.376	0.618 (0.242–0.835)	0.591 (0.172–0.829)	0.0097
Left hippocampus	4362.00 (3993.20, 4784.00)	4066.00 (3801.10, 4371.60)	0.1	0.574 (0.16–0.899)	0.564 (0.132–0.816)	0.0147
Right basal ganglia	46,390.00 (42,911.75, 47,000.00)	46,375.00 (43,834.20, 50,234.80)	0.255	0.084 (− 0.314 to 0.494)	0.146 (− 0.344 to 0.574)	0.5633
Right cerebellum	89,439.00 (84,631.25, 98,000.40)	95,445.00 (89,421.80, 98,207.10)	0.091	0.35 (− 0.06 to 0.681)	0.352 (− 0.138 to 0.703)	0.1521
Right hemisphere	508,069.94 ± 36,007.75	516,248.33 ± 41,078.62	0.53	0.654 (0.298–0.853)	0.68 (0.312–0.871)	0.0019
Right hippocampus	5599.28 ± 777.12	5631.67 ± 531.52	0.885	− 0.004 (− 0.497 to 0.467)	0.009 (− 0.46 to 0.474)	0.971

## Discussions and conclusions

The novelty and potential contributions of our work include the following: 1. The clinical significance of our study is that we used head CT images obtained during PET/CT scans to perform the brain segmentation, which would be an initial yet critical step in the combination of the complementary information provided by PET/CT scans. The usage of high-resolution head CT images ensured accuracy of the extraction of the brain anatomical information. Based on that, functional information from PET images can be analyzed on the segmented brain regions. We thought that this framework might take advantage of each imaging modality and combine the separate information in a more reasonable fashion. 2. Although we didn’t create a novel network architecture, we compared two widely used medical imaging segmentation networks (DenseVNet and 3D U-Net) in performing the segmentation task and presented the results, which might be used as a reference for similar studies. 3. To validate the segmentation results, we specifically enrolled patients who underwent both PET/CT scans and MRI. Then, we used a publicly available MRI atlas method as the reference to provide head-to-head comparisons of the volumetric results as another aspect of the quantitative performance evaluation of the segmentation model.

The mean Dice scores of the eight brain regions segmented by our proposed method were generally high except for left and right hippocampus. Since an independent testing set was used, the Dice results showed robustness of the proposed model. The poor Dice scores of the left and right hippocampus might attribute to three aspects: image quality, size of data set, and parametric settings during training. For image quality, the training and testing data set of 90 patients was obtained with GE Revolution CT scanners (voltage: 120 kVp, current: 225 mAs / 174 mAs) while the independent testing set of 18 patients was obtained with a Siemens BioGraph PET/CT scanner (voltage: 120 kVp, current: 300 mAs). Although appropriate post-processing was applied, image quality differences could result in variations in the segmentation performance. Also, the relatively small size of the training data set might be a limiting factor for the model. Lastly, since our task in this study was to perform multi-label segmentation on head CT, considering the sizes and volumes of all labels, we set the spatial window size as 200 × 200 × 64, which was considerably larger than the hippocampus. Thus, the performance on segmenting the hippocampus might be compromised. Further studies where data sets are of bigger sizes and various spatial window sizes are used can be conducted.

Previous studies focusing on whole brain segmentation on CT images have been sparse, mainly because CT images had poor soft tissue contrast. It was not until recently that more efforts were made on this topic. A recent study by Zhao et al. [6] used a fully convolutional network (FCN) to synthesize MR T1-w images from NCCT images. Then, a standard pipeline of segmentation and labeling was applied on the synthesized T1-w images. The mean Dice scores obtained ranged from 0.51 to 0.73. In our study, four regions of the targeted brain regions obtained Dice scores larger than 0.90, which indicated the advantage of our method, which might be because it performed direct segmentation instead of conducting a synthesizing task first. Cai et al. [24] used a deep learning method to directly segment intracranial structures on brain CT images. Besides a primary dataset that was split into the training, validation, and testing sets. Two secondary datasets were also used to ensure that the model learned generalizable features. Performance metrics were Dice scores, from 0.74 to 0.96, which exceeded those of the existing methods, claimed by the authors. However, the study did not perform a comparison between the segmentation results of the proposed method and those of an MRI-based method.

Our study has the following limitations. Firstly, since CT and MRI are fundamentally different imaging modalities, numerous factors could affect the comparison of voxel-based volumes between the two methods, such as the voxel spacing difference. We tried mitigating the effect by resampling both CT images and MRI to have the same voxel size. Also, we included CT images from separate centers with various imaging parameters. However, further studies focusing on the specific effects of imaging qualities of both CT and MRI can be performed. Secondly, the MRI images used as the reference had a resolution of 0.36 × 0.36 × 6 mm^3^, whose slice thickness was relatively high. This could result in inaccuracy of the segmentation results with the MRI atlas method, especially on fine anatomical regions such as the hippocampus. The poor agreement and correlation results of right hippocampus might be because of this resolution issue. Further studies using MRI images with finer resolution should be conducted. Thirdly, the number of the enrolled patients were limited because it was required that the patients underwent PET/CT, head CT, and MRI so we could conduct head-to-head comparisons of the volumetric results. This limited the generalization of our results. Finally, the number of the brain anatomical regions segmented in our study was relatively small, which was intentionally aligned with the simpler module within the BrainSuite13 software. We thought this work could lay a foundation for future work where a finer segmentation of regions can be investigated.

## Conclusions

Overall, we proposed a deep learning method that used a CNN to perform automated segmentation of brain anatomical regions on head CT images and obtained results from the aspects of independent testing set as well as head-to-head comparisons of region volumes with MRI atlas method. Our results indicate that CT images could be used to provide precise anatomical information of the brain non-inferior to MRI images if proper methods are applied.

## Data Availability

The datasets used and/or analyzed during the current study available from the corresponding author on reasonable request.

## Abbreviations

CT: Computed tomography
PET/CT: Positron emission tomography/computed tomography
MRI: Magnetic resonance imaging
CNNs: Convolutional neural networks
FDG: Fluorodeoxy-glucose
WM: White matter
GM: Grey matter
CSF: Cerebrospinal fluid
TBI: Traumatic brain injury
NCCT: Non-contrast computed tomography
CTP: Computed tomography perfusion
SD: Standard deviation
